# Upstaging of Patients Diagnosed with Favorable Intermediate-Risk Prostate Cancer—Is Active Surveillance Really a Suitable Approach for All These Patients?

**DOI:** 10.3390/cancers17213444

**Published:** 2025-10-27

**Authors:** Analena E. Handke, Christopher Orf, Martina Dellino, Leon Miguel Garcia-Schürmann, Jan Philipp Radtke, Joachim Noldus, Florian Roghmann, Rein-Jüri Palisaar, Sebastian Berg, Karl H. Tully

**Affiliations:** 1 Department of Urology and Neurourology, Marien Hospital Herne, Ruhr-University Bochum, 44801 Bochum, Germany; christopher.orf@elisabethgruppe.de (C.O.); martina.dellino@elisabethgruppe.de (M.D.); leon-miguel.garcia-schuermann@elisabethgruppe.de (L.M.G.-S.); joachim.noldus@elisabethgruppe.de (J.N.); florian.roghmann@elisabethgruppe.de (F.R.); rein-jueri.palisaar@elisabethgruppe.de (R.-J.P.); sebastian.berg@elisabethgruppe.de (S.B.); karl.tully@elisabethgruppe.de (K.H.T.); 2 Department of Urology, University Hospital Duesseldorf, Heinrich-Heine-University Duesseldorf, 40225 Duesseldorf, Germany; janphilipp.radtke@med.uni-duesseldorf.de

**Keywords:** prostate cancer, active surveillance, favorable intermediate-risk, upgrading, upstaging, mpMRI, PI-RADS

## Abstract

Background & Objectives: Current guidelines recognize a subgroup of favorable intermediate-risk (FIR) ISUP grade group (GG) 2 prostate cancer (PCa) that may be eligible for active surveillance (AS). However, upgrading and upstaging to more aggressive disease are frequently observed. We aimed to identify risk factors for adverse pathology in this cohort to better define clinical scenarios where AS may need to be reconsidered. Methods: We retrospectively analyzed 170 patients diagnosed with ISUP GG2 PCa by multiparametric MRI (mpMRI)/TRUS fusion biopsy, all treated with radical prostatectomy (RP). Patients with FIR disease were evaluated for upstaging to ≥pT3 or upgrading to ISUP GG of ≥3 at RP. Multivariable logistic regression identified predictors of adverse pathology. Key Findings and Limitations: Among 170 FIR patients, median PSA was 5.6 ng/mL. Most had PI-RADS 4 (57%) or 5 (20%) lesions; 13% were diagnosed by systematic biopsy only. At RP, 28% showed adverse pathology, including 5 patients (2.9%) with lymph node metastases. Independent predictors were a PI-RADS Score of ≥4, PSA of >7 ng/mL, and clinical T-stage on digital rectal examination. Conclusions and Clinical Implications: Nearly 1/3 of FIR PCa patients were upstaged to high-risk PCa at RP. Based on these findings, AS in clinical practice should only be considered after thorough patient counseling and performed using a stringent follow-up and staging regimen to minimize the risk of further disease progression. A key limitation is the lack of the percentage of Gleason pattern 4.

## Simple Summary

AS is a recommended strategy for low-risk PCa and, more recently, FIR PCa as well. However, concerns remain about the safety of AS for FIR PCa, as some patients may habor more aggressive disease at the time of biopsy. This study analyzed 170 patients diagnosed with FIR PCa to identify predictors of adverse pathology at time of RP. We found that 28% of patients were upstaged to higher-risk categories, with pre-operative PSA levels, PI-RADS Score of ≥4, and clinical T-stage as significant predictors in this highly selective cohort of FIR PCa patients. Our results support AS for carefully selected FIR patients but highlight the need for thorough patient counseling and close follow-up to minimize progression risks. Further research is needed to refine AS criteria and incorporate advanced imaging tools like multiparametric MRI on a general basis.

## 1. Introduction

Active surveillance (AS) has been widely accepted as a recommended treatment strategy for men with low-risk prostate cancer (PCa), with evidence demonstrating equivalent oncological outcomes compared to definitive therapy even at long-term follow-up [1,2,3,4,5]. Data from several prospective trials, including PIVOT and UK ProtecT, demonstrate comparably low prostate cancer-specific mortality for patients with low-risk disease, randomized to active treatment with radical prostatectomy (RP), radiation therapy, or observation at initial PCa diagnosis [6,7].

On this basis, current National Comprehensive Cancer Network (NCCN), American Association of Urology (AUA) and European Association of Urology (EAU) guidelines additionally define a subgroup of favorable intermediate-risk (FIR) ISUP grade group (GG) 2 (PCa), in which AS can be considered a suitable treatment option [8,9]. Historically, these patients usually underwent RP as the primary course of treatment. However, based on the recommendations of the Canadian consensus group, ASCO and the DETECTIVE study consensus, AS and deferred treatment can also be considered for patients with favorable ISUP grade group 2 PCa (e.g., PSA < 10 ng/mL, low density, clinical stage ≤ cT2a and a low number of positive systematic cores) [10,11].

Despite this shift, concerns remain. In daily clinical practice, a proportion of FIR patients are found to harbor more aggressive disease at final pathology, raising questions about the safety of surveillance in this setting. Published data on AS outcomes for intermediate-risk PCa are inconsistent, with some studies suggesting acceptable cancer-specific outcomes and others highlighting risks of adverse reclassification during follow-up [12,13]. This ongoing controversy underscores the clinical challenge of identifying which FIR patients are suitable for AS and which are at risk of progression if treatment is deferred.

Therefore, the present study aimed to examine histopathologic grade and stage in this specific subgroup of patients and to determine the proportion with unfavorable intermediate- or high-risk disease at the time of RP in a larger unicentric cohort. We further aimed to identify predictors of adverse pathology to refine patient selection for AS among FIR cases.

## 2. Materials and Methods

### 2.1. Patient Inclusion

We retrospectively collected and analyzed a prospective database of 473 patients from our high-volume institution. These patients were diagnosed with ISUP GG 2 PCa by MRI/TRUS fusion targeted (TB) and systematic biopsy (SB) by transrectal or transperineal approach by software-guided biopsy between July 2021 and October 2023, who all underwent RP between September 2021 and September 2024. Eligible patients had ≤ than three positive biopsy cores, PCa-infiltration in less than 50%, a clinical T-stage ≤ cT2a and a PSA-value ≤ 10 ng/mL. The given criteria were based on the NCCN guideline. All investigators had experience with more than 50 biopsies and were supervised by physicians who had performed over 350 procedures. Multiparametric MRIs (mpMRI) were performed using PI-RADS v2.1 [14]. If mpMRIs were performed externally, the internal uro-radiologists performed a follow-up assessment prior to the MRI/TRUS fusion biopsy and mapped the region of interest. No patients received neoadjuvant therapy before RP. All patients were treated with robotic-assisted or retropubic RP. Histopathology was reviewed according to clinical routine by expert dedicated urogenital pathologists. The institutional ethics committee approved this study (Approval number: 2024-469-f-S).

### 2.2. Outcome Measurements and Predictors

Adverse outcomes (AOs) were defined as ISUP upgrading (ISUP ≥ 3) or adverse pathology upstaging (AP = pT3 at RP and/or pN1) in the final RP histopathology. Patients were stratified according to NCCN definition as favorable intermediate-risk (FIR) PCa with ≤3 biopsy cores positive for ISUP GG 2 PCa and overall PCa detection in less than 50% of all biopsy cores, ≤cT2a disease in digital rectal examination (DRE), and a prostate-specific antigen (PSA) of <10 ng/mL. We further assessed parameters like age, biopsy approach, prostate volume, PSA, PI-RADS Score and number of lesions, the maximum number of cores and number of positive cores, ISUP of each core in TB as well as SB, percentage of infiltration of positive cores and analyzed them as variables predicting AO. From the RP specimen, we assessed TNM, residual tumor classification, ISUP GG, surgical approach, and whether or not lymph node dissection (LND) was performed.

### 2.3. Statistical Analysis

Medians, interquartile ranges (IQRs), frequencies, and proportions were generated for continuous and categorical variables. We conducted multivariable logistic regression models to calculate the odds of upstaging accounting for pre-biopsy multiparametric MRI results, pre-operative PSA, clinical T-stage, prostate volume, and proportion of positive biopsies. All analyses used Stata (Version Stata/SE 15.1, Stata Corp LLC, College Station, TX, USA). Results were considered statistically significant for *p* < 0.05.

## 3. Results

### 3.1. Baseline Characteristics

**Figure 1:**
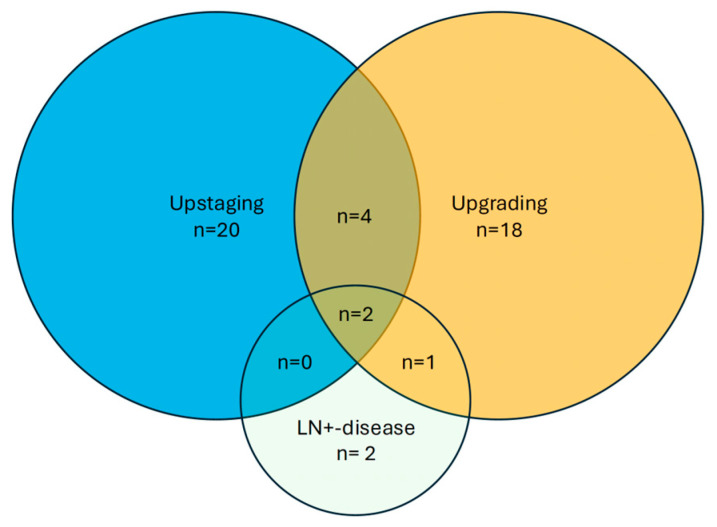
Venn diagram of patients being upstaged to an unfavorable risk strata based on tumor grade, tumor stage, or lymph-node positive disease.

Overall, 473 patients were diagnosed with ISUP GG 2 PCa at MRI/TRUS fusion biopsy, and 170 (37%) were classified as FIR PCa. Patient baseline characteristics are shown in Table 1 with a median PSA at diagnosis of 5.6 ng/mL (Interquartile range (IQR) 4.7 ng/mL; 7.2 ng/mL). Most patients presented with PI-RADS 4 (*n* = 96; 57%) or PI-RADS 5 (*n* = 34; 20%) lesions at pre-biopsy mpMRI, while 13% (*n* = 22) of these patients had negative mpMRI and were identified by systematic biopsy alone. The median time from histological diagnosis to surgery was 72 days (IQR 56; 91 days). Interestingly, no patients in our cohort were classified as unfavorable intermediate-risk PCa. However, five patients (2.9%) were upstaged because of lymph node-positive disease. Altogether, 28% of cases (*n* = 47) were upstaged to high-risk PCa at the time of RP. A detailed distribution of patients who were upstaged to an unfavorable risk-stratification is portrayed in Figure 1.

### 3.2. Uni- and Multivariable Regression Analyses to Identify Predictors of Upstaging/Upgrading Possibility

On uni- and multivariable analysis, a PI-RADS lesion ≥ 4 was a significant predictor for the upstaging/upgrading of FIR PCa patients as shown in Table 2, respectively (Odds ratio (OR) 2.61, 95% Confidence interval (CI) 1.02–6.71, *p* = 0.046; OR 2.93, 95% CI 1.08–7.93, *p* = 0.034). On univariable analysis, an increasing PSA remains insignificant. On multivariable analysis, pre-operative PSA with a threshold above 7 ng/mL was also an independent predictor (OR 1.19, 95% CI 0.98–1.46, *p* = 0.076; 1.30, 95% CI 1.06–1.60, *p* = 0.013). Moreover, clinical T-stage (i.e., cT2a vs. T1c) (OR 2.65, 95% CI 1.18–5.93, *p* = 0.018; OR 3.68, 95% CI 1.52–8.93, *p* = 0.004) was a significant predictor of upstaging/upgrading at the time of surgery in FIR PCa (Table 2). Prostate volume and the proportion of positive cores at the time of biopsy showed no significant association regarding upstaging and upgrading in patients undergoing RP for FIR PCa. A receiver-operating characteristics analysis (ROC) examining the predictive value of the logistic regression model showed an area under the curve (AUC) of 0.71 (Figure S1).

## 4. Discussion

In this cohort from a high-volume center, 28% of men with MRI/TRUS fusion biopsy diagnosed with FIR PCa were upgraded on RP, i.e., 28% harbored disease of ISUP ≥ 4 with even 2.9% lymph node-positive stage. An increasing PSA, higher PI-RADS, and a clinical T-stage were significant predictors for upgrading to ISUP ≥ 3, pT ≥ 3, or AP, with clinical T-stage as the strongest predictor.

The debate surrounding AS in intermediate-risk PCa stems mainly from variations in definitions and guideline recommendations for managing this disease [15]. According to the EAU guidelines, AS should be considered for patients with ISUP GG 2 disease, characterized by, for example, <10% Gleason pattern 4, PSA < 10 ng/mL, ≤cT2a, low disease extent on imaging, and limited tumor involvement in biopsies, defined as ≤3 positive cores with an ISUP GG 2 and ≤50% cancer involvement per core or another isolated feature of intermediate-risk disease combined with low disease extent in imaging and biopsies. In contrast, the NCCN guidelines offer a more individualized and even less restrictive approach, i.e., patients with ≥3 positive cores with an ISUP GG 2 and ≤50% cancer involvement per core can be considered for AS. According to the current German S3 guideline, patients with a PSA level above 15 ng/mL, a cT2c palpation finding, cribriform and ductal growth, or a higher proportion of Gleason pattern 4 should not be considered for active surveillance [16].

The reported incidence of upgrading varies significantly across studies. In a cohort of over 10,000 patients with FIR PCa, Yang et al. observed a 33% incidence of upgrading or upstaging [17]. Another study that included intermediate-risk disease found AP outcomes in 36% of patients [18]. However, Ploussard et al. reported a 25% rate of Gleason score upgrading in men with biopsy GG and cT1–2 stage [19]. Similarly, Gandaglia et al. identified unfavorable disease as non-organ-confined or ISUP ≥ 3 disease at RP specimen in 33% of patients within a cohort of low- and intermediate-risk individuals [20]. The proportion of upgraded patients observed in our study (i.e., ca. 28% of patients) aligns with these findings. In a recent publication by Baboudjian et al., 46% of patients who underwent RP were upgraded/upstaged with GG ≥ 3 and/or pT3a, but the very aggressive disease (GG 4 and/or pT3b and/or pN1) was observed in only 7% [12]. Our results are comparable with 7,6% upgrading/upstaging (GG ≥ 4 and/or pT3b).

It should be noted, however, that biopsy techniques varied considerably among the cited studies. Given the growing adoption of fusion biopsy in recent years, it is likely that the majority of cases involved standard systematic biopsy. Improved imaging and biopsy techniques may account for the reduced rates of upgrading observed in final pathology. This was corroborated by a recent multicenter study comparing upgrading rates following TB versus SB [21]. It was shown that upgrading and AP outcomes depend on the biopsy method. FIR disease identified solely via TB was most concordant with RP findings and presented a significantly lower risk of upgrading compared to ISUP 2 disease detected through both TB and SB. Two additional studies emphasized that in the era of pre-biopsy mpMRI and image-guided biopsies, traditional criteria like “number of positive cores” are less relevant. Many patients who fail to meet these criteria may still have a low absolute risk of local or distant progression [20,21,22]. The DETECTIVE study consensus highlighted that when mpMRI-based TB is performed, the number of positive cores is not a reliable indicator of disease extent or tumor volume. Instead, tumor volume may be inferred from the number and length of cancerous cores in systematic biopsies or the volume of the dominant lesion seen on mpMRI [10]. Furthermore, MRI and TB are likely to identify small foci of Gleason grade 4 cancer that might be missed by systematic biopsy.

Currently, international guidelines recommend AS be offered to FIR patients with low tumor burden, defined as a maximum of three cores with ISUP GG 3 + 4 [22]. Nonetheless, prior studies have identified other influencing factors, including age, PSA levels, clinical T stage, number and percentage of positive cores, tumor involvement per core, or perineural invasion [18,19,20,23,24]. PI-RADS has also been recognized as a predictor for upgrading to ISUP GG ≥ three and AP, as reported in a single institution study by Pham et al. [23]. Additionally, lesion volume has been shown to predict upgrading to ISUP ≥ 3 in FIR PCa. Klotz et al. observed that candidates with better prognoses included men with intermediate-risk disease characterized by a PSA level of 10–20 ng/mL, ISUP GG 2 disease with a small proportion of Gleason pattern 4, and either negative mpMRI findings or negative targeted biopsy results [2]. Our findings support these observations, as the absolute number of positive cores was not associated with an increased risk of upgrading. Instead, we identified that elevated PSA levels and PI-RADS 4-5 lesions on mpMRI significantly correlated with a higher likelihood of upgrading and upstaging. These results are consistent with two previous studies, which identified these factors as critical in selecting the most suitable candidates for AS [21,25].

Nevertheless, regarding new pre-operative staging modalities, e.g., PSMA-PET/CT, the importance of PLND in FIR PCa patients should be questioned individually. One solution to this could be risk models, such as the Briganti score, which indicates the risk of lymph node metastasis so that additional patient selection could be generated [26]. However, such an application still appears underrepresented in everyday clinical practice.

In addition to clinical and imaging parameters, genomic and molecular biomarkers have shown promise in further refining AS eligibility and predicting reclassification risk in intermediate-risk PCa. Although not routinely recommended in current guidelines, their incorporation into individualized decision-making may enhance risk stratification and optimize the safety of AS in selected cases [27,28,29].

Some limitations should be acknowledged. First, the study lacks information on the proportion of the Gleason 4 pattern per core, which is currently required to classify FIR according to EAU guidelines. Second, its retrospective design may have introduced unmeasured confounders. Third, the relatively small sample size limits the statistical power of subgroup analyses. Fourth, while a general limited LND field was used for all these patients (i.e., surgical removal of the nodal packet under the external iliac vein and above and below the obturator nerve), certain aspects, such as prior surgeries and a certain degree of surgeon variability, may introduce some inter-surgeon variability regarding PLND, for which we cannot account for in our analyses. Similarly, potential variability arises from differences in biopsy approaches and from the fact that multiple physicians were involved in performing both biopsies and radical prostatectomies, which may have introduced heterogeneity in sampling, diagnostic accuracy, and surgical technique. Finally, a possible selection bias should be acknowledged in this highly selective single center cohort of FIR PCa patients. Future studies may validate the given results in an AS-managed cohort.

## 5. Conclusions

AS is a safe strategy for well-selected patients with FIR PCa. Based on our findings, AS should be offered only after comprehensive patient counseling and implemented within a rigorous follow-up and staging framework to minimize the risk of disease progression. Future studies and guidelines are crucial for specifying the inclusion criteria and developing a personalized, risk-based AS protocol incorporating mpMRI findings.

## Figures and Tables

**Table 1:** Baseline and tumor-specific characteristics of patients diagnosed with favorable intermediate-risk prostate cancer undergoing radical prostatectomy (*n* = 170).

**(a) Pre-Operative Characteristics**		
Median age at the time of diagnosis, years (IQR)		65 (59, 70)
Median PSA * at time of diagnosis, ng/mL (IQR)		5.6 (4.7, 7.2)
Median prostate volume, ccm (IQR)		36 (28.1, 50)
Digital rectal examination (clinical T-Stage), *n* (%)	T1	139 (81.8)
	T2a	31 (18.2)
Highest PI-RADS * lesion at pre-biopsy MRI *, *n* (%)	≤2	22 (13.0)
	3	18 (10.6)
	4	96 (56.5)
	5	34 (20.0)
Biopsy approach, *n* (%)	transrectal	96 (56.5)
	transperineal	74 (43.5)
**(b) Surgical Characteristics**		
Median time to surgery, days (IQR)		72 (56, 91)
Surgical approach	Open	5 (2.9)
	Robotic	165 (97.1)
Pathologic T-Stage	T2	144 (84.7)
	T3a	18 (10.6)
	T3b	8 (4.7)
Pathologic N-Stage	N0	165 (97.1)
	N+	5 (2.9)
Pathologic R-Stage	R0	168 (98.8)
	R1	2 (1.2)
Final Gleason Score	7a	145 (85.3)
	7b	24 (14.1)
	8	0 (0)
	9	1 (0.6)
**(c) Postoperative Complications**		
Postoperative Lymphocele, *n* (%)	Yes	25 (14.7)
	No	145 (85.3)
Venous compression, *n* (%)	Yes	6 (3.5)
	No	164 (96.5)
Fever, *n* (%)	Yes	7 (4.1)
	No	163 (95.9)
Drainage necessary, *n* (%)	Yes	12 (7.1)
	No	158 (92.9)

* MRI, magnetic resonance imaging; PSA, prostate specific antigen; PI-RADS, Prostate Imaging Reporting and Data System; Gleason 7a, ISUP 2; Gleason 7b, ISUP 3; Gleason 8, ISUP 4; Gleason 9, ISUP 5.

**Table 2:** Uni- and multivariable analysis examining pre-operative predictors of upstaging and upgrading in patients undergoing radical prostatectomy for favorable intermediate prostate cancer.

	Univariable Analysis			Multivariable Analysis		
Variable	OR *	95% CI *	*p*-Value	OR *	95% CI *	*p*-Value
PI-RADS Score ≥ 4	2.61	1.02–6.71	0.046	2.93	1.08–7.93	0.034
Pre-operative PSA	1.19	0.98–1.46	0.076	1.30	1.06–1.60	0.013
Clinical T-stage	2.65	1.18–5.93	0.018	3.68	1.52–8.93	0.004
Prostate Volume	0.99	0.97–1.01	0.411	0.99	0.97–1.01	0.208
Proportion of positive cores at the time of biopsy	0.84	0.38–18.4	0.911	0.18	0.06–5.10	0.312

* CI, Confidence Interval; OR, Odds ratio.

## Data Availability

The original contributions presented in this study are included in the article/Supplementary Materials. Further inquiries can be directed at the corresponding author(s).

## Supplementary Materials

The following supporting information can be downloaded at: https://www.mdpi.com/article/10.3390/cancers17213444/s1, Figure S1: Study flow diagram displaying the assembly of the study cohort of patients with favorable intermediate risk prostate cancer undergoing radical prostatectomy between September 2021 and September 2024.
