# Supplementary material for: Upstaging of Patients Diagnosed with Favorable Intermediate-Risk Prostate Cancer—Is Active Surveillance Really a Suitable Approach for All These Patients?
Source: Cancers (Basel). 2025 Oct 27;17(21):3444. doi: 10.3390/cancers17213444 (PMC12607737; doi:10.3390/cancers17213444)
Supplement: Supplementary file 1 [file cancers-17-03444-s001.zip › cancers-3914931-supplementary.pdf]

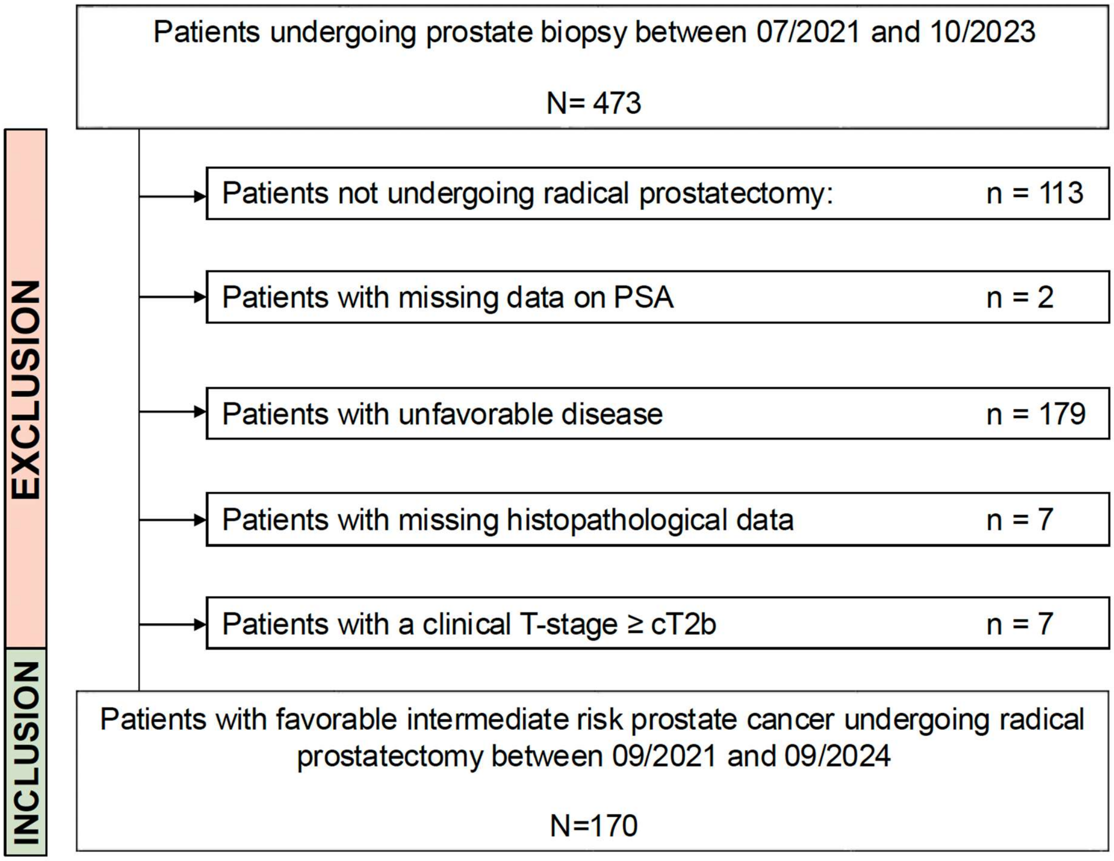


**Figure S1**. Study flow diagram displaying the assembly of the study cohort of patients with favorable intermediate risk prostate cancer undergoing radical prostatectomy between 09/2021 and 09/2024
